# The association of caffeine consumption with positive affect but not with negative affect changes across the day

**DOI:** 10.1038/s41598-025-14317-0

**Published:** 2025-08-05

**Authors:** Justin Hachenberger, Yu-Mei Li, Anu Realo, Sakari Lemola

**Affiliations:** 1 Department of Psychology, Bielefeld University, Universitätsstraße 25, 33615 Bielefeld, Germany; 2 Medical School OWL, Bielefeld University, Bielefeld, Germany; 3 Department of Psychology, University of Warwick, Coventry, UK; 4 Institute of Psychology, University of Tartu, Tartu, Estonia; 5 School of Educational Sciences, Tallinn University, Tallinn, Estonia

**Keywords:** Caffeine, Affect, Experience sampling methodology, Human behaviour, Psychology

## Abstract

Caffeine is well known for its stimulant effects on the central nervous system, leading to enhanced cognitive performance and changes in affective states. While these effects have been extensively studied in controlled laboratory settings, caffeine’s influence on affect in everyday life remains comparatively underexplored. This study aims to bridge this gap by examining the associations between momentary caffeine consumption and affective states in naturalistic settings, while also accounting for potential moderators such as time of the day, individual differences, and contextual factors. Employing an experience sampling methodology (ESM), we analyzed data from 115 participants aged 18–25 in Study 1 and 121 participants aged 18–29 in Study 2. Study 1 lasted 14 days yielding 8335 completed surveys and Study 2 lasted 28 days yielding 19,960 completed surveys. Our findings indicate that caffeine intake was associated with subsequent increases in positive affect, while associations with decreases in negative affect were less consistent. The association between caffeine consumption and positive affect was strongest within the first 2.5 h after awakening (i.e., in the morning). Tiredness and social context moderated this association. Overall, the findings of this ESM study suggest that caffeine may play an important role in modulating affective states in everyday life.

## Introduction

Caffeine is a central nervous system stimulant that is used by approximately 80% of the world’s population^1,2^ and consumed in a wide variety of forms, including beverages like coffee, tea, soda (e.g., coke), and energy drinks, but also in food items such as chocolate. Positive expectations related to caffeine consumption include reduced tiredness and fatigue, enhanced cognitive and physical performance, and a positive shift in mood^3–8^. While there is a large body of evidence for such beneficial effects coming from experimental studies^2,9–18^, there is a lack of studies investigating associations of caffeine particularly with mood or affective states in naturalistic, real-world settings. Therefore, the current study focuses on the momentary associations between caffeine consumption and affective states in daily life. Additionally, we examine potential moderators of this association, including time of day, individual differences (e.g., mental health, sleep quality, habitual caffeine use), and contextual factors (e.g., tiredness, social setting, type of day) which are often overlooked or difficult to account for in controlled laboratory settings.

The stimulant effect of caffeine is assumed to be due to its property as an adenosine antagonist^9,19,20^. Adenosine inhibits the central nervous system by reducing the release of stimulating and activating neurotransmitters (e.g., dopamine or noradrenaline) and also plays a role in the aetiology of depressive and anxiety disorders^21^. Caffeine blocks the receptors of adenosine and thereby increases the release of dopamine and noradrenaline^9,19,20^. Due to its wake-promoting qualities related to blocking adenosine receptors, caffeine may have stronger effects on mood in the morning compared to later in the day as it may help people jump-starting the day and overcoming sleep inertia^22^. However, to the best of our knowledge, systematic research examining whether the relationship between caffeine consumption and affective states varies across different times of day (i.e., elapsed time after awakening, clock time, or time since one’s sleep midpoint) remains limited.

The effects of caffeine on affect may vary substantially between individuals. For example, it has been claimed that the beneficial effects of caffeine are only caused by a reversal of withdrawal symptoms^9,23,24^. Some studies have found mood improvements only in habitual caffeine consumers^25,26^. However, the evidence is mixed with mood improvements also being observed in non-consumers and in habitual consumers who were not exposed to a withdrawal^9–11^. Beyond the question of withdrawal reversal, it has been reported that caffeine consumption may lead to increased anxiety and jitteriness, especially when high doses were consumed^10,27,28^. These findings have been interpreted as over-stimulation of the central nervous system to which individuals prone to anxiety and depression may be particularly sensitive. In an ecological momentary assessment study, Whalen et al.^29^ for instance found that adolescents with major depression showed more anxiety or nervousness on days when they consumed caffeine. Another potential negative effect of caffeine consumption may be related to sleep disturbance, which may in particular be caused by caffeine consumption later in the day^30^. In sum, some individuals appear to be particularly sensitive to excessive caffeine consumption due to individual differences in caffeine metabolism^27^ or mental health^28,31^ for whom negative effects of caffeine consumption may predominate. By contrast, caffeine consumption seems to enhance positive emotions in those individuals who are habitual consumers of caffeine due to offsetting of withdrawal symptoms. These individual differences highlight the need to account for person-specific factors when examining how caffeine consumption relates to affective states in everyday life.

Other potential moderators of the effects of caffeine on mood may involve psychophysiological states or contextual factors, including, for instance, whether caffeine is consumed together with other people or alone. Various studies using experience sampling methodology (ESM) have shown that such factors are related to affective wellbeing independent of caffeine consumption^32–38^. For instance, perceived effects of caffeine on mood may be more strongly influenced by social context—such as whether coffee is consumed during a break with colleagues or alone—rather than solely by caffeine’s chemical effects on the brain. These factors have not been studied in previous research, specifically in daily settings.

### Aims of the present study

Overall, the current state of research on the relationship between caffeine consumption and mood is subject to some limitations.

First, most research on the associations of caffeine consumption with mood outcomes has been conducted in laboratory settings under controlled environments^2,9,10^ and therefore, the results of these studies have low ecological validity.

Second, many previous studies refer to “mood” when examining the emotional effects of caffeine, while they actually assess relatively immediate, reactive states which would be more accurately described as affect^39,40^. Generally, mood refers to a more persistent and sustained emotional state while affects tend to be more short-termed states that are reactive to either internal or external stimuli^39,40^. In particular, research that distinguishes between positive and negative affect^39^ following caffeine consumption is scarce. In the present study we focus on the effect of caffeine consumption on people’s affective states. We are interested in both general positive and negative affective states as well as in more specific affects subsumed under positive and negative affect. Therefore, we also analyze single affect items separately in order to differentiate between positive and negative affect items with varying degrees of activation or arousal according to the Circumplex Model of Affective States^41^.

Third, aside from some anecdotal reports and non-systematic research^22^, there is a lack of studies examining whether the relationship between caffeine consumption and affect varies depending on the time of day (i.e., elapsed time after awakening, clock time, or elapsed time since one’s sleep midpoint). A closer investigation of how potential caffeine effects of affect change throughout the day is warranted.

Fourth, the potential roles of psychophysiological states (e.g., current level of tiredness), contextual factors (e.g., being around other people), and individual differences in caffeine sensitivity (e.g., mental disorders, sleep quality) in the relationship between caffeine consumption and affect have been rarely studied. Therefore, the present study aims to investigate how these factors moderate the relationship between caffeine consumption and subsequent positive and negative affective states. In summary, the overarching aim of this study is to examine whether caffeine consumption is related to changes in affective experience and whether this relationship is moderated by various daytime-related, biological, individual, and social factors in everyday life, using an ESM with high ecological validity^42^. Specifically, the study addresses four research questions:

- (RQ1) To what extent is the consumption of caffeinated beverages related to positive and negative affect?
- (RQ2) Whether and to what extent does the relationship between caffeine consumption and affect vary across the day (i.e., by the elapsed time since awakening, clock time, or elapsed time since sleep midpoint)?
- (RQ3) Whether and to what extent does this relationship differ among individuals with varying levels of caffeine sensitivity, such as those with different levels of depressive symptoms, anxiety symptoms, sleep quality, caffeine dependency or expected withdrawal symptoms, and habitual caffeine consumption?
- (RQ4) To what extent do psychophysiological states (e.g., level of tiredness) and contextual factors (e.g., social setting, work day versus day off) moderate the relationship between caffeine consumption and affect?

These research questions will be studied in two convenience samples of young adults below 30 years of age, while adjusting for time-varying covariates including sleep duration of the previous night and sleep midpoint, which served as indicators for sleep timing. Based on previous research we hypothesized that

#### Hypothesis 1

The consumption of caffeinated beverages is associated with higher subsequent positive (1a) and lower negative affect (1b).

#### Hypothesis 2

The associations between caffeine consumption and positive (2a) and negative (2b) affect are strongest shortly after waking up (i.e., in the morning) because of caffeine’s potential of jump-starting wake-promoting mechanisms and overcoming sleep inertia. While this hypothesis primarily focuses on elapsed time since awakening, we will also investigate clock time and elapsed time since sleep midpoint as potential moderators in sensitivity analyses, as these variables likely overlap substantially but not completely.

No hypotheses are formulated for the third and fourth research questions due to a lack of prior studies or inconsistent findings in existing research. Instead, the effects of psychophysiological states, contextual factors, and individual differences will be investigated on an exploratory basis.

## Methods

### Design and procedure

Data from two ESM studies were used to address the research questions of the present study. Both studies were approved by the Ethics Committee of Bielefeld University (reference number 2020-138 and 2022-063). All procedures were in accordance with the Declaration of Helsinki and its later amendments. The datasets have been used in previous studies^34,37,43,44^, but have not been analyzed for the present purpose. Data collection occurred in two batches from March to April 2021 for Study 1 and in two batches from April to July 2022 for Study 2. When registering for the study, participants were informed about the procedure and conditions of study participation as well as their rights (e.g., regarding data protection and withdrawal from the study). All participants provided informed consent and confirmed that they met the following prerequisite criteria: (a) being 18 to 25 years old in Study 1 and 18 to 29 years old in Study 2. Further inclusion criteria comprised (b) fluency in German and (c) having a smartphone with an Android operating system available for the duration of the study. The latter criterion applied only to Study 1, as participants in Study 2 could use a lab phone to participate.

For each batch, data collection lasted 15 days in Study 1 and 29 days in Study 2. On the first day, participants completed a baseline questionnaire. On the second day, ESM surveys presented on movisensXS (movisens GmbH, Karlsruhe, Germany) started. Participants were asked to complete short surveys seven times per day for 14 consecutive days in Study 1 and for 28 consecutive days in Study 2.

In Study 1, participants received a prompt for the first survey of the day between 8:00 am and 9:00 am on weekdays and between 9:00 am and 10:00 am on weekends. For the next five signal-contingent surveys during the day, prompts were sent out between 10:30 am and 7:00 pm on weekdays and between 11:30 am and 7:00 pm on weekends. Each day’s last prompt was sent out between 9:00 pm and 10:00 pm on both weekdays and weekends.

In Study 2, participants were able to manually start the first survey of each day and were asked to do so as soon as possible after waking up. If participants did not start the first survey manually, they received a reminder prompt between 8:00 am and 9:00 am on weekdays and between 9:00 am and 10:00 am on weekends. For the next five surveys during the day, prompts were sent out between 10:30 am and 7:00 pm. Each day’s last prompt was sent out between 9:00 pm and 10:00 pm on weekdays and weekends.

In both studies, all prompts were sent out at random times within the respective time interval. Also, there was a gap of at least 90 min between two adjacent prompts. Participants could respond to the surveys within 30 min of receiving the prompts. If participants did not respond, the survey was automatically marked as missing. Participants were instructed to ignore prompts in situations that could pose danger to themselves or others (e.g., while driving).

### Participants

#### Study 1

A total of 158 individuals participated in Study 1. Of these, 43 were excluded because they did not report having consumed caffeinated beverages at any time when sleep data of the preceding night was also available. The final sample consisted of 115 participants (98 females, 16 males, and one person did not indicate their gender; mean age = 22.8 years, *SD* = 1.9) providing 6,122 responses with complete data required for the analyses in this study. On average, participants in the final sample provided complete data for 53.2 assessment points (*SD* = 19.4, range = 5–83).

#### Study 2

A total of 134 individuals participated in Study 2. Of these, 13 were excluded because they did not report having consumed caffeinated beverages at any time when sleep data of the preceding night was also available. The final sample consisted of 121 participants (104 females, 15 males, and two persons indicated their gender as diverse; mean age = 23.8 years, *SD* = 2.8) providing 17,204 responses with complete data required for the analyses in this study. On average, participants in the final sample provided complete data for 142.2 assessment points (*SD* = 40.0, range = 7–194).

### Measures and instruments

The instruments used to measure the relevant constructs and variables were largely identical in both studies and are therefore described together. Any differences between the two studies are explicitly noted.

#### Baseline questionnaire

##### Typical daily caffeine intake

In Study 1, participants were asked about their habitual consumption of a variety of caffeinated beverages (coffee, espresso, black/green tea, coke, energy drinks). For each caffeinated beverage, participants indicated how many cups/cans/bottles they typically consume in one day. Reference values for typical volumes were given for each beverage.

In Study 2, participants were asked about their habitual consumption of a variety of caffeinated beverages such as coffee, espresso, black tea, green tea, coke, and energy drinks. For each caffeinated beverage, participants indicated how many millilitres (ml) they typically consume in one day.

In order to harmonize the responses for the different beverages and to calculate a measure for the typical daily caffeine intake, the data for each beverage was converted into the corresponding caffeine concentration based on values provided by the European Food Safety Authority^45^. The caffeine concentration per beverage were then summed to determine the typical daily caffeine intake of each participant.

##### Caffeine dependency

In Study 1, caffeine dependency was measured with three items from the Dependency subscale of the German version of the Caffeine Expectancy Questionnaire (CaffEQ)^5,7^. The three items were (1) “I would experience caffeine withdrawal if I went without caffeine”, (2) “I need to have caffeine every day”, and (3) “I would get a headache if I went without caffeine”. Participants were asked to indicate on a visual analogue scale how likely each statement would apply to them (0 = *very unlikely*; 10 = *very likely*). Out of the three items, a sum score was computed. The internal consistency of the scale was α = 0.91.

In Study 2, the complete 12-item Dependency subscale of the CaffEQ^5,7^ was used. Participants were asked to indicate on a 6-point scale how likely each statement would apply to them (1 = *very unlikely*; 6 = *very likely*). Out of all items, a sum score was computed. The internal consistency of the subscale was α = 0.87.

Schott et al.^7^ reported moderate to high correlations of the CaffEQ dependency subscale with other caffeine-related variables such as average caffeine intake, caffeine use disorder symptoms, and caffeine dependence related symptoms, indicating evidence of convergent validity. In our study, the CaffEQ dependency subscale was moderately correlated with the typical daily caffeine intake (*r* = 0.49, *p* < 0.001).

##### Depressive symptoms

Depressive symptoms were measured at baseline with the Depressiveness subscale (PHQ-9) of the German version of the Patient Health Questionnaire (PHQ-D)^46,47^. The participants indicated on a 4-point scale (0 = *not at all*, 1 = *several days*, 2 = *more than half the days*, 3 = *nearly every day*) how often the nine depressive symptoms occurred in the preceding 1 week (Study 1) or two weeks (Study 2). Out of all nine items, a sum score was computed (0–27 points). The internal consistency reliability of the subscale was α = 0.81 in Study 1 and α = 0.83 in Study 2.

##### Anxiety symptoms

Anxiety symptoms were measured at baseline with the subscale for Generalized Anxiety Disorder (GAD-7) of the PHQ-D^46,48^. The participants indicated on a 4-point scale (0 = *not at all*, 1 = *several days*, 2 = *more than half the days*, 3 = *nearly every day*) how often the anxiety symptoms occurred in the preceding 1 week (Study 1) or two weeks (Study 2). Out of all seven items, a sum score was computed (0–21 points). The internal consistency of the GAD-7 was α = 0.83 in Study 1 and α = 0.87 in Study 2.

##### Sleep quality

Sleep quality was measured at baseline with the German version of the Pittsburgh Sleep Quality Index (PSQI)^49^. The PSQI is a 19-item, self-rated questionnaire designed to measure sleep quality in the past month. The 19 items are divided into seven components, including sleep duration, sleep disturbance, sleep latency, disruption of daytime functioning due to sleepiness, sleep efficiency, overall sleep quality and use of sleep medications. Each of the sleep components results in a score between 0 and 3. The values of the sleep components are summed together to a total score (0–21 points), with a higher score indicating poorer sleep quality (see^49^ for more details).

#### Experience sampling method surveys

##### Caffeine consumption

In each ESM survey (except the first survey of each day in Study 1 as explained earlier), the participants were asked whether they had consumed caffeinated beverages within the preceding 90 min (“Have you consumed any caffeinated beverages during the last 90 min?”; responses: *no*/*yes*).

##### Positive and negative affect

The measurement of affect was based on Das-Friebel et al.^50^ and Lenneis et al.^51^ but was modified in some important respects (i.e., we reduced the number of items and used a different response scale). In our study, participants were asked to indicate on a visual analogue scale how much they felt each emotion at the given moment by responding to the question, “How … do you feel at the moment?” (0 = *not at all*, 100 = *very much*).

Positive affect was measured with the items *content*, *enthusiastic*, and *happy*. Negative affect was measured with the items *sad*, *upset*, and *worried*. Sum scores for positive and negative affect were computed with higher scores indicating higher levels of positive and negative affect, respectively. The internal consistency for the positive affect score across all measurement points was ω_between_ = 0.93 and ω_within_ = 0.76 in Study 1 and ω_between_ = 0.95 and ω_within_ = 0.81 in Study 2. The internal consistency for the negative affect score was ω_between_ = 0.91 and ω_within_ = 0.65 in Study 1 and ω_between_ = 0.88 and ω_within_ = 0.66 in Study 2. The internal consistency coefficients were computed using R-package *multilevelTools*^52^.

##### Tiredness

To measure momentary tiredness, participants were asked to indicate on a visual analogue scale how tired they currently feel (“How tired do you feel at the moment?”, 0 = *not at all*, 100 = *very much*).

##### Being with other people

In Study 1, the participants were asked in all surveys except the first survey of each day about their company at the moment (“Who is with you right now (including video calls)?”). If the participant indicated that they were alone, it was classified as “no” for “being with other people”, otherwise it was classified as “yes”. In Study 2, participants were asked in the second to sixth daily survey whether they were currently with other people (“Are there other people with you right now?”; responses: no/yes).

##### Workday vs. free day

In both studies, the participants were asked in the first survey of each day whether the respective day was a workday for them or not (“Is today a regular work/university day for you?”; responses: *no*/*yes*).

##### Sleep duration and sleep midpoint

N both studies, the participants provided a sleep diary in the first survey of each day. The participants were asked at what time they went to sleep ("When did you turn off the light and go to sleep?”), how long it took them to fall asleep ("How long did it take you to fall asleep last night?"), and at what time they woke up (“What time did you wake up this morning?”). Based on these items, we calculated the sleep duration and the sleep midpoint.

### Statistical analyses

Data preprocessing and all statistical analyses were performed using R (version 4.3.2)^53^. All hypotheses were tested with multilevel models using the R-package *lme4* (version 1.1-31)^54^. Restricted maximum likelihood estimation was used to obtain parameters of the multilevel models. All models included a random intercept and a random slope for the main predictor caffeine consumption (see more detailed model specifications and equations in Supplement 1). Last night’s sleep duration and sleep midpoint as indicators for sleep timing were included as covariates in all analyses.

To test the association of momentary reports of caffeine consumption and positive and negative affect (RQ1/Hypothesis 1), a dichotomous variable indicating recent caffeine consumption was included as a predictor and momentary ratings of (a) positive and (b) negative affect as outcome variables, respectively. Also, the single affect items (content, enthusiastic, happy, sad, upset, and worried) were investigated as outcomes.

To investigate changes across the day in the associations between momentary reports of caffeine consumption and affect (RQ2/Hypothesis 2), we categorized the ESM surveys into six time frames based on the elapsed time since awakening when the survey was started. The time frames were: (1) 0–2.5 h, (2) 2.5–5 h, (3) 5–7.5 h, (4) 7.5–10 h, (5) 10–12.5 h, and (6) more than 12.5 h after awakening.

These time frames were selected to ensure sufficient observations and thus adequate statistical power per category, while also maintaining the resolution as fine as possible. Multilevel models testing the interaction between momentary reports of caffeine consumption and time frame on affective states were computed, followed by simple slope analyses using the R-package *interactions* (version 1.1.5)^55^ for each time frame.

For further sensitivity analyses, we also investigated clock time and elapsed time since sleep midpoint as moderators. For both variables, the 2.5-h time frames were retained for categorization. For clock time, these were: (1) before 9:30 am, (2) 9:30 am–12:00 pm, (3) 12:00 pm–2:30 pm, (4) 2:30 pm–5:00 pm, (5) 5:00 pm–7:30 pm, and (6) after 7:30 pm. For elapsed time since the sleep midpoint, the time frames were: (1) 0–5 h, (2) 5–7.5 h, (3) 7–5–10 h, (4) 10–12.5 h, (5) 12.5–15 h, and (6) more than 15 h after sleep midpoint. To test moderating effects of individual differences in caffeine sensitivity (i.e., depressive and anxiety symptoms, caffeine dependency, typical caffeine intake, and sleep quality on the relationship between momentary ratings of caffeine consumption and affect; RQ3), the models testing the association between the momentary reports of caffeine consumption and affect (RQ1) were extended by a cross-level interaction between caffeine consumption and caffeine dependency (CaffEQ), typical daily caffeine intake, depressive symptoms (PHQ-9), anxiety symptoms (GAD-7), and sleep quality (PSQI). The effect of each moderator was examined in a separate model.

To examine psychophysiological states and contextual factors as moderators (RQ4), the models testing the association between momentary reports of caffeine consumption and affect (RQ1) were extended by adding an interaction term between momentary caffeine consumption and (a) current level of tiredness (*t*), (b) level of tiredness reported at the prompt before (*t*-1) (c) whether the participant was around other people, and (d) whether it was a workday or not. The effects of moderators were examined in separate models. The level of tiredness and whether the participant was around other people could vary within days while whether it was a workday or not could only vary between days.

To account for within-individual effects^56^ and to facilitate interpretation and comparison of effects, all continuous predictors or moderators assessed with ESM (i.e. tiredness) were within-individual standardized (using the individual means and standard deviations), while the outcome variables (i.e., affective states) and all continuous predictors assessed in the baseline questionnaire (i.e. typical daily caffeine intake, caffeine dependency, depressive symptoms, anxiety symptoms, and sleep quality) were grand-mean standardized (using the group-mean and standard deviation) before the analysis. A significance threshold of α = 0.05 was used. To address the problem of alpha error accumulation in multiple comparisons, all *p*-values were adjusted by using false discovery rate (FDR)^57^ within each study.

## Results

### Descriptive statistics

Demographics and descriptive statistics of both studies are displayed in Table 1. In an average of 8.6 surveys (16.2%) in Study 1 and 23.8 surveys (16.7%) in Study 2, participants reported having consumed caffeinated beverages during the preceding 90 min of completing the survey.

**Table 1:** Sample characteristics and descriptive statistics.

	Study 1 (*n* = 115)		Study 2 (*n* = 121)	
	*n* (%)/*M* (*SD*)	Min–Max	*n* (%)/*M* (*SD*)	Min–Max
Baseline				
Gender				
Female	98 (85.2)	–	104 (86)	–
Male	16 (13.9)	–	15 (12.4)	–
Diverse or missing	1 (0.9)	–	2 (1.7)	–
Age	22.8 (1.9)	18–25	23.8 (2.8)	18–29
Anxiety symptoms (GAD-7)	7.1 (4.5)	0–20	6.8 (4.6)	0–21
Depressive symptoms (PHQ-9)	8.0 (4.9)	0–22	8.3 (4.7)	1–24
Sleep quality (PSQI)	5.2 (2.5)	1–13	6.5 (3.1)	1–16
Caffeine dependency (CaffEQ)	7.2 (9.0)	0–30	25.7 (15.3)	12–68
Typical daily caffeine intake [mg]	120.7 (95.7)	0–395	142.2 (153.8)	0–833
Experience sampling				
Completed questionnaires	53.2 (19.4)	5–83	142.2 (40)	7–194
Reported caffeine consumption	8.6 (7.8)	1–42	23.8 (16.8)	1–76
Positive affect	58.2 (13.8)	19.5–86.9	54.5 (12.8)	25.4–80.8
Content	63.8 (14.5)	27.8–92.9	58.6 (13.4)	29.2–85.5
Enthusiastic	46.8 (15.5)	3.9–87.4	46.5 (13.5)	11.7–79
Happy	63.9 (14.9)	23.5–94.1	58.3 (13.6)	31–86
Negative affect	19.8 (13.2)	0.7–56.6	23.4 (13.1)	1.6–59.6
Sad	17.9 (13.7)	0–55.2	20.6 (13.9)	0.5–65.9
Upset	15.6 (12.5)	0.7–60.3	18.3 (12.5)	1.1–58.1
Worried	25.9 (17.2)	0.6–85.8	31.4 (17)	2.2–69.3
Tiredness	43.5 (15.4)	2.5–82.3	46.1 (13.7)	14.9–80.4
Reported being around others	28.4 (15.4)	3–66	45.5 (21.1)	0–94
Questionnaires on workdays	21.7 (17.5)	0–64	84.9 (36.6)	0–152
Sleep duration [h]	7.4 (1.2)	3.5–10.2	7.3 (0.9)	4.0–10.0
Sleep midpoint [hh:mm]	04:17 (00:52)	02:23–07:17	04:01 (00:50)	00:42–06:19

*GAD-7* generalized anxiety disorder-7, *PHQ-9* patient health questionnaire-9, *PSQI* Pittsburgh sleep quality index, *CaffEQ* caffeine expectancy questionnaire.

### Caffeine consumption and subsequent affect

Multilevel models examining RQ1 showed that momentary reports of caffeine consumption were associated with higher momentary positive affect in both studies compared to when no caffeine consumption was reported (*Study 1*: β = 0.08, *p* < 0.05; *Study 2*: β = 0.14, *p* < 0.001). Hypothesis 1a was thus confirmed. The association of caffeine consumption with positive affect had a standard deviation of 0.18 (95%-CI[0.10, 0.26]) in Study 1 and 0.14 (95%-CI[0.08, 0.19]) in Study 2, indicating that the association significantly varies between individuals and supporting further analyses of moderating role of individual differences.

The single item analyses revealed that consuming beverages containing caffeine was associated with feeling enthusiastic (*Study 1*: β = 0.12, *p* < 0.01; *Study 2*: β = 0.15, *p* < 0.001) in both studies. A significant association with feeling happy (β = 0.12, *p* < 0.001) and content (β = 0.10, *p* < 0.001) was only observed in Study 2.

Multilevel models testing Hypothesis 1b showed that caffeine consumption was associated with lower negative affect in Study 2 (β = –0.08, *p* < 0.001), but not in Study 1. The single item analyses revealed that caffeine consumption was associated with feeling less sad (β = –0.10, *p* < 0.001) and less upset (β = –0.08, *p* < 0.001) in Study 2, but not in Study 1. Feeling worried was not significantly related with caffeine consumption in either study. All results for RQ1 were displayed in Supplementary Table 1.

### Changes in the caffeine-affect relationship depending on the time elapsed since awakening

**Fig. 1:**
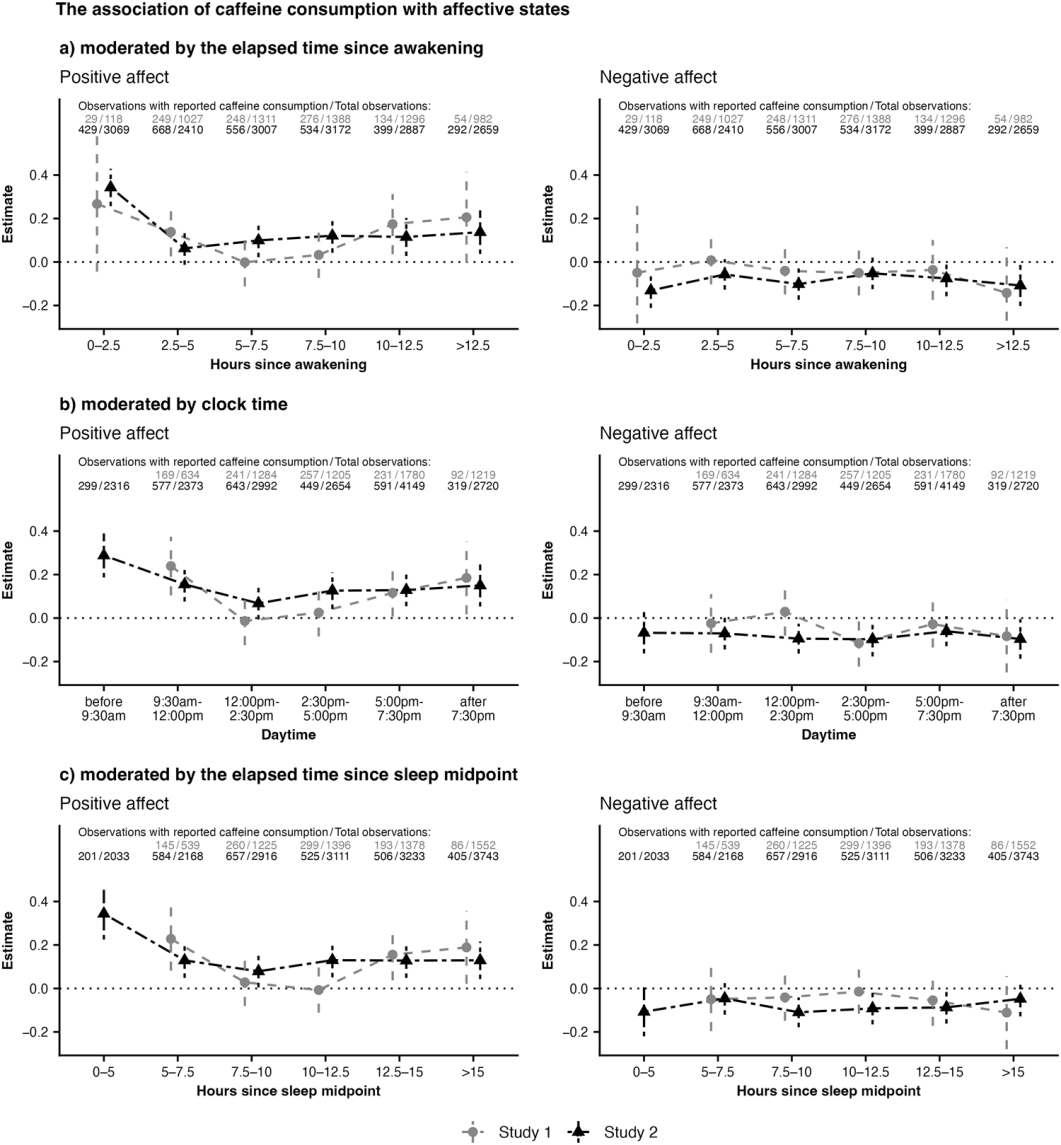
Changes of the association between caffeine consumption and affective states across the day. This figure demonstrates the beta coefficients of the associations between caffeine consumption and subsequent affective states across the day. Vertical lines represent 95% confidence intervals.

The analyses of the association between caffeine consumption and positive affect across the elapsed time since awakening examining RQ2 (see Fig. 1a) revealed that the association was strongest in the first 2.5 h after awakening (*Study 1*: β = 0.27, *p* = 0.184; *Study 2*: β = 0.34, *p* < 0.001). Afterwards the strength of the associations decreased and was no longer significant (i.e., 2.5–5 h after awakening in *Study 2*: β = 0.06 *p* = 0.151, or 5–7.5 h after awakening in *Study 1*: β = 0.00, *p* = 0.989). The strength of the association increased again slightly towards the evening (i.e., 10–12.5 h after awakening; *Study 1*: β = 0.17, *p* < 0.05; *Study 2*: β = 0.12, *p* < 0.05). This is further supported by the comparison between later time frames to the first time frame (i.e., 0–2.5 h after awakening) in Study 2: the association between caffeine consumption and positive affect was weaker in all later time frames compared to the first time frame (see Supplementary Table 2 for the results). Hypothesis 2a was thus supported.

There were no specific changes in the association between caffeine consumption and negative affect depending on the elapsed time since awakening in either study, and therefore, Hypothesis 2b was not supported.

Sensitivity analyses investigating clock time and elapsed time since sleep midpoint as moderators for the caffeine-affect relationship showed similar patterns and were displayed in Fig. 1b,c. All results for RQ2 were displayed in Supplementary Table 2.

### The role of caffeine sensitivity on the relationship between momentary ratings of caffeine consumption and affect

Next, we investigated whether and to what extent the relationships between momentary ratings of caffeine consumption and positive and negative affect vary across groups with different levels of caffeine sensitivity, including factors such as caffeine dependency/expected withdrawal symptoms, habitual caffeine consumption, depressive symptoms, anxiety symptoms, and sleep quality (RQ3).

No cross-level-interaction between momentary ratings of caffeine consumption and baseline ratings of caffeine dependency (CaffEQ score), typical daily caffeine intake, depressive symptoms (PHQ-9 score), anxiety symptoms (GAD-7 score), or sleep quality (PSQI score) on affective states were found. All results for RQ3 were displayed in Supplementary Table 3.

### Analyses of psychophysiological states and contextual factors as moderators

When investigating psychophysiological states and contextual factors as moderators (RQ4) of the relationship between momentary ratings of caffeine consumption and positive affect, we found that the associations of caffeine consumption with positive affect were moderated by current tiredness (β = 0.06, *p* < 0.01), tiredness reported in the preceding momentary survey (β = 0.04, *p* < 0.05), and being around other people (β = –0.11, *p* < 0.01) in Study 2. These findings indicate that individuals who were more tired than usual reported greater increases in positive affect following caffeine consumption, whereas the association between caffeine consumption and positive affect was weaker when individuals were around other people. No significant moderation by workday vs. free day was found.

Concerning negative affect, no moderating associations of the tested variables were found. All results for RQ4 were displayed in Supplementary Table 4.

## Discussion

The present study aimed to explore the relationship between momentary caffeine consumption and affect in everyday life, while also considering the role of various potential moderating factors such as time of day (i.e., elapsed time since awakening, clock time, and elapsed time since the sleep midpoint), individual differences in caffeine sensitivity, and contextual factors. Our research contributes to the existing literature with four key findings. First, we provided supporting evidence that caffeine consumption is associated with elevated affect, particularly with higher positive affect (Hypothesis 1a). This association was also consistently present for individual positive affect items—namely, feeling enthusiastic and content. The relationship between higher caffeine consumption and lower negative affect was observed only in Study 2 (Hypothesis 1b). While the associations with individual negative affect items were also inconsistent. Second, as hypothesized, the association of caffeine consumption and positive affect was the strongest shortly after waking up (Hypothesis 2a), while no differences across the day were found for negative affect (Hypothesis 2b). Third, the associations of caffeine consumption and affective states were not moderated by individual differences such as depressive symptoms, anxiety symptoms, sleep quality, typical daily caffeine intake or caffeine dependency (RQ3). Fourth, Study 2 provided evidence that psychophysiological states, such as feeling tired and contextual factors, such as being in the company of others, can play a role in the effects of caffeine consumption (RQ4).

### Differences in caffeine’s association with positive and negative affect

Consistent with Hypothesis 1a, we found that momentary caffeine consumption was associated with higher levels of positive affect as well as with individual positive affect items. This supports previous research suggesting that caffeine has mood-enhancing effects^9,11–18^. On the contrary, findings for Hypothesis 1b were less consistent. While in Study 2, caffeine consumption was associated with lower negative affect, particularly with feeling less sad and upset, these associations were not found in Study 1. In addition, the effect size for the relationship between caffeine consumption and negative affect was considerably smaller than for positive affect. This is in line with studies examining other factors (e.g., physical activity) for subsequent affect that find inconsistent or weaker effects for negative affect in contrast to positive affect^58^. Negative affect could be more closely tied to enduring personal or contextual factors (e.g., experiencing stressful periods or impactful life events), which are not as readily influenced by the acute effects of caffeine or other factors. This could explain why the reduction in negative affect does not follow the same mechanisms as the increase in positive affect observed with caffeine consumption.

### Changes of the caffeine-affect relationship depending on the elapsed time since awakening

Findings related to Hypothesis 2a provide insights into how the elapsed time since awakening moderates the relationship between caffeine consumption and increases in positive affect. Our data suggest that the affect-enhancing effects of caffeine are most pronounced within the first 2.5 h after waking, indicating a strong temporal dimension to caffeine’s benefits for positive affect. This pattern was further supported by sensitivity analyses investigating clock time and elapsed time since sleep midpoint which showed similar results. The mechanism behind this morning accentuation could be threefold.

First, morning caffeine consumption may align with habitual use patterns, where the anticipation of caffeine’s effects contributes to its affect-enhancing properties^8^. Psychological factors, such as the expectation of positive effects and the ritualistic aspects of consuming coffee or tea after awakening, could amplify its impact on positive affect. This suggests that the relationship between caffeine consumption and positive affect may not be purely biological but also influenced by psychological factors.

Second, past research has found no clear dose–response relationship between caffeine and subsequent mood or cognitive performance^9^. Instead, the initial consumption of caffeine appears to induce these effects, while additional intake does not produce further benefits, at least not within eight hours of the first consumption^9,59^. This interpretation aligns with the withdrawal reversal hypothesis, which posits that the benefits of caffeine, especially in habitual users, mainly reflect the alleviation of withdrawal symptoms that build up during overnight abstinence^24,25^. From this perspective, the pronounced effect after awakening may result from reversing withdrawal-related low arousal or mood. Since many people typically consume their first coffee or tea in the morning (e.g., at breakfast), the effect could therefore be strongest at this time of day.

Third, the morning accentuation of the caffeine-affect relationship aligns with theories about caffeine’s blockade of adenosine receptors and its interactions with circadian rhythms^19–21,60,61^. Caffeine may act as a zeitgeber^62^, an external timing signal involved in the entrainment of circadian rhythms^63^—which is the process of synchronizing the internal biological clock involving rhythms with the external light–dark cycle—and in adjusting the amplitude of biological rhythms. The influence of zeitgebers is stronger when they occur closer to the circadian nadir, which typically happens in the middle of the main sleep period^64^. Thus, the circadian modulation of caffeine’s effects may be more pronounced when consumption occurs near the main sleep period. In the morning, caffeine may help compensate for reduced cortisol secretion in habitual consumers^65^ by supporting wake-promoting mechanisms through activation of the sympathetic nervous system, thereby boosting energy and enhancing positive affect.

### The role of individual differences as moderators

The exploration of individual differences in caffeine sensitivity (RQ3) revealed no moderating effects of caffeine dependence, typical daily caffeine intake, depressive symptoms, anxiety symptoms, or sleep quality on the relationship between momentary ratings of caffeine consumption and affect. One possible explanation for the absence of moderation effects could be that individuals who are particularly sensitive to caffeine and may react negatively to it avoid consuming caffeine. These individuals would have been excluded from our analysis, as a prerequisite for inclusion was that caffeine consumption was reported at least once. Of course, this does not apply to individuals with high values for caffeine dependency and high typical consumption, as it is to be expected that they, by definition, report caffeine consumption more frequently. However, past research yielded inconsistent evidence for differences between low vs high habitual consumers. Some studies only found mood improvements in habitual caffeine consumers^25,26^, while others observed mood improvements in low habitual consumers who were not exposed to a withdrawal^11^. Possible differences between low and high habitual users may have been distorted in our study because people with higher typical caffeine intake may consume caffeine more frequently throughout the day, while people with low-to-moderate caffeine intake may only consume caffeine once a day. As discussed above, past research has suggested that only the first consumption, particularly in the morning, shows significant effects^9,59^, so that the association could be statistically reduced in high-frequency consumers, although the first consumption may be stronger than in low-to-moderate consumers. A specific investigation of the first caffeine consumption of the day and an implementation of an intervention would, therefore, be of particular interest in future research.

### Psychophysiological states and contextual factors as moderators

Our exploration of psychophysiological and contextual factors (RQ4) revealed that the levels of tiredness and social context moderated the effects of caffeine consumption on affect. The increased benefit for positive affect in Study 2 when individuals were more tired than usual aligns with caffeine’s known stimulant effects, providing empirical support for its use as a countermeasure to tiredness. Furthermore, in Study 2 we found that the associations of caffeine consumption and positive affect diminished when individuals were in the company of others. This suggests that social contexts may dilute the perceived affect benefits of caffeine, possibly due to the social interactions themselves impacting current affect or altering the individual’s awareness of caffeine’s effects in contrast to situations in which individuals are alone and maybe focus on studying or work.

### Inconsistencies between the two studies

The results of the two studies differed in several respects. First, single item analyses revealed that feeling content and happy were predicted by caffeine consumption only in Study 2. Second, a significant association between caffeine consumption and negative affect, as well as related single items, was found only in Study 2. Third, differences in the relationship between caffeine and positive affect at various times after waking were significant only in Study 2. Fourth, significant moderation by psychophysiological and contextual factors (i.e., tiredness and social situation) was observed exclusively in Study 2.

The most reasonable explanation for these inconsistencies is the difference in the number of sampled measurement occasions, which affected the statistical power to detect smaller effects. Study 2 included considerably more observations, as it was conducted over a four-week period. Supporting this interpretation, effect sizes in Study 1 were mostly similar to those in Study 2, despite the lack of statistical significance.

Another possible explanation relates to the differing circumstances under which the two studies were conducted. Study 1 took place during the peak phase of a widespread COVID-19 lockdown in Germany, which had a significant impact on everyday life and severely restricted social interactions. In contrast, Study 2 was conducted during a period when some measures remained in place, but were much milder in terms of restrictions^66^.

### Limitations and future research

While this study contributes valuable insights into the relationship between caffeine consumption and affect in everyday life, it is necessary to be aware of some limitations. First, the reliance on self-reported measures introduces the potential for bias, and the observational nature of the study limits causal inferences. Second, the sample consisted solely of young adults, which limits the generalizability of the findings to other age groups. Furthermore, the sample is gender biased, as a significantly higher number of females participated. Third, the investigation of depressive symptoms and anxiety symptoms assessed with a screening questionnaire as moderators for the association between caffeine and affect is not comparable or generalizable to clinical diagnoses. Fourth, we did not assess the exact timing of the caffeine consumption, the preceding presence of potential withdrawal symptoms, or whether a consumption was the first or subsequent. Fifth, we did not directly assess participants’ chronotype, which could have provided additional insights into circadian influences on our findings.

In terms of future research, a more detailed assessment of the contextual aspects of caffeine consumption would be particularly valuable for studying its effects of affective states. Specifically, the exact timing of caffeine consumption, the presence of withdrawal symptoms before the consumption, and whether the consumption is the first of the day should be investigated in greater detail. Furthermore, device-based measurements of circadian rhythm, such as skin temperature and heart rate to determine the individual circadian time of caffeine consumption, would be of great interest for future research.

## Conclusion

Taken together, this study provides evidence that caffeine consumption is associated with increases in momentary positive affect in everyday life, with effects being particularly pronounced shortly after waking up (i.e., in the morning). However, the relationship between caffeine and affect was not moderated by individual differences in caffeine sensitivity, but rather by factors such as tiredness and social context.

## Supplementary Information

Supplementary Information.

## Data Availability

The datasets used and/or analysed during the current study as well as the analysis code are available from the corresponding author on reasonable request.
