# Supplementary material for: The association of caffeine consumption with positive affect but not with negative affect changes across the day
Source: Sci Rep. 2025 Aug 5;15:28536. doi: 10.1038/s41598-025-14317-0 (PMC12325641; doi:10.1038/s41598-025-14317-0)
Supplement: Supplementary file 1 — Supplementary Information. [file 41598_2025_14317_MOESM1_ESM.pdf]

Supplementary Material

The Association of Caffeine Consumption With Positive Affect but Not With Negative Affect Changes Across the Day

Justin Hachenberger[1,*], Yu-Mei Li[1,2], Anu Realo[3,4,5], and Sakari Lemola[1]

[1]Department of Psychology, Bielefeld University, Bielefeld, Germany

[2]Medical School OWL, Bielefeld University, Bielefeld, Germany

[3]Department of Psychology, University of Warwick, Coventry, United Kingdom

[4]Institute of Psychology, University of Tartu, Tartu, Estonia

[5]School of Educational Sciences, Tallinn University, Tallinn, Estonia

Supplement 1

Equations for the Multilevel Models Used for the Statistical Analyses

Research Question 1

Multilevel models testing Hypotheses 1a/1b (RQ1) were defined by the following equations:

Level-1:

$$Y_{ij} = \beta_{0j} + \beta_{1j} * Caffeine\ Consumption_{ij} + Covariates + r_{ij}$$

Level-2:

$$\beta_{0j} = \gamma_{00} + u_{0j}$$

$$\beta_{1j} = \gamma_{10} + u_{1j}$$

The within-subject effects were estimated at level 1. The subscript j refers to participant j and subscript i to the time of measurement. Affect (positive affect, negative affect, and single items) at time i in participant j is represented by Y_{ij}. The intercept and the effects of caffeine consumption are represented by β at level 1. The residuals are represented by r_{ij}. At level 2 we included a random intercept and a random slope for caffeine consumption. u_{0j} represents the variation of participants' intercept around the mean intercept (γ_{00}). u_{1j} represent the variation of participants' individual slopes for caffeine consumption around the respective overall mean slope (γ_{10}).

Research Questions 2 and 4

Multilevel models testing Hypotheses 2a/2b (RQ2) and RQ4 were defined by the following equations:

Level-1:

$$Y_{ij} = \beta_{0j} + \beta_{1j} * Caffeine\ Consumption_{ij} + \beta_{2j} * Moderator_{ij} +$$

$$\beta_{3j} * Caffeine\ Consumption_{ij} * Moderator_{ij} + Covariates + r_{ij}$$

Level-2:

$$\beta_{0j} = \gamma_{00} + u_{0j}$$

$$\beta_{1j} = \gamma_{10} + u_{1j}$$

$$\beta_{2j} = \gamma_{20}$$

$$\beta_{3j} = \gamma_{30}$$

The within-subject effects were estimated at level 1. The subscript j refers to participant j and subscript i to the time of measurement. Affect (positive affect, negative affect) at time i in participant j is represented by Y_{ij}. The intercept and the effects of caffeine consumption, the respective moderator (time of day, tiredness, being around other people, freeday vs workday), and the respective interaction term between caffeine consumption and moderator are represented by β at

level 1. The residuals are represented by r_{ij}. A random intercept was included. We only considered a random slope for caffeine consumption as the inclusion of random slopes for the moderator variables and respective interaction term lead to non-convergence of the models in a high proportion of cases. u_{0j} represents the variation of participants' intercept around the mean intercept (γ_{00}). u_{1j} represent the variation of participants' individual slopes for caffeine consumption around the respective overall mean slope (γ_{10}). γ_{20} and γ_{30} represent fixed slope for the moderator variable and the interaction term, respectively.

Research Question 3

Multilevel models (including a cross-level interaction) testing RQ3 were defined by the following equations:

Level-1:

$$Y_{ij} = \beta_{0j} + \beta_{1j} * Caffeine\ Consumption_{ij} + Covariates + r_{ij}$$

Level-2:

$$\beta_{0j} = \gamma_{00} + u_{0j}$$

$$\beta_{1j} = \gamma_{10} + \gamma_{11} * Baseline\ Moderator_j + u_{1j}$$

The within-subject effects were estimated at level 1. The subscript j refers to participant j and subscript i to the time of measurement. Affect (positive affect, negative affect) at time i in participant j is represented by Y_{ij}. The intercept and the effects of caffeine consumption are represented by beta β at level 1. The residuals are represented by r_{ij}. A random intercept was included. We only considered a random slope for caffeine consumption. u_{0j} represents the variation of participants' intercept around the mean intercept (γ_{00}). u_{1j} represent the variation of participants' individual slopes for caffeine consumption around the respective overall mean slope (γ_{10}). γ_{11} represents the coefficient for the cross-level interaction.

Supplementary Table 1

Main Results (RQ1/Hypothesis 1)

	Study 1			Study 2		
Outcome	Beta	*CI*	*p*	Beta	*CI*	*p*
Positive affect	0.08	0.02 – 0.15	**< .05**	0.14	0.10 – 0.19	**< .001**
Content	0.05	-0.01 – 0.12	.211	0.10	0.06 – 0.14	**< .001**
Enthusiastic	0.12	0.05 – 0.19	**< .01**	0.15	0.11 – 0.20	**< .001**
Happy	0.04	-0.03 – 0.11	.441	0.12	0.08 – 0.17	**< .001**
Negative affect	-0.03	-0.09 – 0.03	.492	-0.08	-0.12 – -0.04	**< .001**
Sad	-0.05	-0.12 – 0.01	.168	-0.10	-0.14 – -0.06	**< .001**
Upset	-0.01	-0.08 – 0.05	.873	-0.08	-0.12 – -0.05	**< .001**
Worried	-0.01	-0.08 – 0.05	.880	-0.02	-0.06 – 0.01	.277

Supplementary Table 2

Time Moderators (RQ2/Hypothesis 2)

Moderator		Study 1			Study 2		
		Beta	*CI*	*p*	Beta	*CI*	*p*
Positive affect							
Time frames	Caffeine	0.27	-0.04 – 0.58	.184	0.34	0.26 – 0.43	**< .001**
	TF2	0.15	-0.01 – 0.31	.130	0.21	0.16 – 0.26	**< .001**
	TF3	0.22	0.06 – 0.38	**< .05**	0.20	0.16 – 0.24	**< .001**
	TF4	0.22	0.06 – 0.38	**< .05**	0.19	0.15 – 0.23	**< .001**
	TF5	0.21	0.05 – 0.36	**< .05**	0.23	0.19 – 0.27	**< .001**
	TF6	0.20	0.04 – 0.36	**< .05**	0.23	0.18 – 0.27	**< .001**
	Caffeine:TF2	-0.13	-0.45 – 0.20	.643	-0.28	-0.39 – -0.17	**< .001**
	Caffeine:TF3	-0.27	-0.59 – 0.06	.207	-0.24	-0.35 – -0.14	**< .001**
	Caffeine:TF4	-0.23	-0.56 – 0.09	.292	-0.22	-0.33 – -0.11	**< .001**
	Caffeine:TF5	-0.09	-0.43 – 0.25	.782	-0.23	-0.34 – -0.11	**< .001**
	Caffeine:TF6	-0.06	-0.43 – 0.31	.880	-0.21	-0.33 – -0.08	**< .01**
Negative affect							
Time frames	Caffeine	-0.05	-0.37 – 0.27	.880	-0.13	-0.21 – -0.05	**< .01**
	TF2	0.08	-0.08 – 0.25	.492	-0.00	-0.05 – 0.04	.917
	TF3	0.10	-0.06 – 0.27	.371	-0.00	-0.04 – 0.04	.965
	TF4	0.06	-0.10 – 0.23	.640	-0.01	-0.05 – 0.03	.724
	TF5	0.05	-0.12 – 0.21	.778	-0.04	-0.08 – 0.00	.128
	TF6	0.05	-0.11 – 0.22	.732	-0.03	-0.07 – 0.01	.223
	Caffeine:TF2	0.06	-0.28 – 0.39	.880	0.07	-0.03 – 0.18	.215
	Caffeine:TF3	0.01	-0.33 – 0.34	.989	0.03	-0.08 – 0.13	.655
	Caffeine:TF4	-0.00	-0.33 – 0.33	.989	0.08	-0.03 – 0.18	.193
	Caffeine:TF5	0.01	-0.33 – 0.36	.979	0.06	-0.06 – 0.17	.393
	Caffeine:TF6	-0.10	-0.48 – 0.28	.800	0.02	-0.10 – 0.14	.764

Supplementary Table 3

Baseline Moderators (RQ3)

		Study 1			Study 2		
Moderator		Beta	*CI*	*p*	Beta	*CI*	*p*
Positive affect							
PHQ-9	Caffeine	0.08	0.02 – 0.15	**< .05**	0.14	0.10 – 0.19	**< .001**
	PHQ-9	-0.30	-0.42 – -0.18	**< .001**	-0.29	-0.39 – -0.18	**< .001**
	Caffeine:PHQ-9	-0.01	-0.07 – 0.06	.940	-0.03	-0.07 – 0.02	.257
GAD-7	Caffeine	0.08	0.02 – 0.15	**< .05**	0.14	0.10 – 0.19	**< .001**
	GAD7	-0.24	-0.36 – -0.11	**< .01**	-0.21	-0.32 – -0.10	**< .001**
	Caffeine:GAD-7	0.02	-0.05 – 0.08	.812	-0.00	-0.05 – 0.04	.918
PSQI	Caffeine	0.08	0.02 – 0.15	**< .05**	0.14	0.10 – 0.19	**< .001**
	PSQI	-0.16	-0.29 – -0.03	**< .05**	-0.15	-0.26 – -0.03	**< .05**
	Caffeine:PSQI	-0.02	-0.09 – 0.05	.790	0.01	-0.04 – 0.06	.694
CaffEQ	Caffeine	0.09	0.02 – 0.15	**< .05**	0.15	0.11 – 0.20	**< .001**
	CaffEQ	-0.14	-0.27 – -0.01	.073	-0.07	-0.19 – 0.04	.252
	Caffeine:CaffEQ	-0.01	-0.07 – 0.06	.923	-0.04	-0.08 – 0.01	.135
TDCI	Caffeine	0.09	0.02 – 0.15	**< .05**	0.15	0.11 – 0.20	**< .001**
	TDCI	-0.03	-0.16 – 0.10	.842	-0.06	-0.18 – 0.05	.336
	Caffeine:TDCI	-0.01	-0.07 – 0.06	.940	-0.04	-0.08 – 0.00	.104
Negative affect							
PHQ-9	Caffeine	-0.03	-0.09 – 0.03	.492	-0.08	-0.12 – -0.05	**< .001**
	PHQ9	0.31	0.18 – 0.43	**< .001**	0.33	0.23 – 0.44	**< .001**
	Caffeine:PHQ-9	-0.00	-0.06 – 0.06	.989	0.02	-0.02 – 0.05	.429
GAD-7	Caffeine	-0.03	-0.09 – 0.03	.477	-0.08	-0.12 – -0.04	**< .001**
	GAD7	0.34	0.22 – 0.46	**< .001**	0.37	0.26 – 0.47	**< .001**
	Caffeine:GAD-7	-0.01	-0.07 – 0.05	.880	-0.00	-0.04 – 0.04	.902
PSQI	Caffeine	-0.03	-0.09 – 0.03	.492	-0.08	-0.12 – -0.04	**< .001**
	PSQI	0.17	0.03 – 0.30	**< .05**	0.16	0.04 – 0.28	**< .05**
	Caffeine:PSQI	0.01	-0.05 – 0.08	.871	0.00	-0.03 – 0.04	.859
CaffEQ	Caffeine	-0.03	-0.09 – 0.03	.474	-0.08	-0.12 – -0.05	**< .001**
	CaffEQ Depend	0.04	-0.09 – 0.18	.732	0.01	-0.11 – 0.14	.864
	Caffeine:CaffEQ	0.01	-0.05 – 0.07	.880	0.02	-0.02 – 0.05	.423
TDCI	Caffeine	-0.03	-0.09 – 0.03	.492	-0.08	-0.12 – -0.05	**< .001**
	TDCI	0.04	-0.09 – 0.18	.732	0.02	-0.10 – 0.15	.754
	Caffeine:TDCI	0.00	-0.06 – 0.06	.989	0.01	-0.03 – 0.05	.674

Note. TDCI typical daily caffeine intake.

Supplementary Table 4

Psychophysiological and Contextual Moderators (RQ4)

		Study 1			Study 2		
Moderator		Beta	*CI*	*p*	Beta	*CI*	*p*
Positive affect							
Tiredness	Caffeine	0.03	-0.03 – 0.10	.463	0.06	0.02 – 0.10	**< .01**
	Tiredness	-0.21	-0.23 – -0.19	**< .001**	-0.31	-0.32 – -0.29	**< .001**
	Caffeine:Tiredness	0.00	-0.05 – 0.05	.967	0.05	0.02 – 0.08	**< .01**
Tiredness-Lag	Caffeine	0.11	0.04 – 0.18	**< .05**	0.12	0.08 – 0.17	**< .001**
	Tiredness-Lag	-0.13	-0.15 – -0.11	**< .001**	-0.17	-0.18 – -0.15	**< .001**
	Caffeine:Tiredness-Lag	0.02	-0.03 – 0.07	.705	0.04	0.01 – 0.08	**< .05**
Around others	Caffeine	0.11	0.02 – 0.19	**< .05**	0.14	0.08 – 0.19	**< .001**
	Around others	0.33	0.29 – 0.37	**< .001**	0.33	0.30 – 0.36	**< .001**
	Caffeine:Around others	-0.06	-0.17 – 0.04	.417	-0.11	-0.18 – -0.04	**< .01**
Workday	Caffeine	0.09	0.01 – 0.17	.072	0.16	0.10 – 0.22	**< .001**
	Workday	-0.08	-0.13 – -0.04	**< .01**	-0.17	-0.20 – -0.14	**< .001**
	Caffeine:Workday	-0.02	-0.13 – 0.09	.880	-0.03	-0.10 – 0.04	.427
Negative affect							
Tiredness	Caffeine	0.01	-0.05 – 0.06	.940	-0.04	-0.07 – -0.00	.051
	Tiredness	0.11	0.09 – 0.13	**< .001**	0.16	0.15 – 0.17	**< .001**
	Caffeine:Tiredness	0.06	0.01 – 0.11	.076	-0.03	-0.06 – 0.01	.163
Tiredness-Lag	Caffeine	-0.04	-0.10 – 0.03	.449	-0.09	-0.13 – -0.04	**< .001**
	Tiredness-Lag	0.08	0.06 – 0.10	**< .001**	0.10	0.08 – 0.11	**< .001**
	Caffeine:Tiredness-Lag	0.01	-0.04 – 0.06	.880	-0.02	-0.06 – 0.01	.224
Around others	Caffeine	-0.06	-0.14 – 0.02	.312	-0.07	-0.13 – -0.02	**< .05**
	Around others	-0.24	-0.28 – -0.19	**< .001**	-0.18	-0.22 – -0.15	**< .001**
	Caffeine:Around others	0.06	-0.04 – 0.17	.428	0.00	-0.07 – 0.07	.995
Workday	Caffeine	-0.06	-0.14 – 0.01	.188	-0.09	-0.15 – -0.04	**< .01**
	Workday	0.11	0.06 – 0.16	**< .001**	0.08	0.05 – 0.11	**< .001**
	Caffeine:Workday	0.08	-0.02 – 0.19	.239	0.02	-0.04 – 0.09	.509
